# Autophagy and Its Regulators in Response to Stress in Plants

**DOI:** 10.3390/ijms21238889

**Published:** 2020-11-24

**Authors:** Wanlong Su, Yu Bao, Xiaoqian Yu, Xinli Xia, Chao Liu, Weilun Yin

**Affiliations:** 1 Beijing Advanced Innovation Center for Tree Breeding by Molecular Design, Beijing Forestry University, Beijing 100083, China; wanlongsu@163.com (W.S.); byaq0556@gmail.com (Y.B.); yuxiaoqian1209@163.com (X.Y.); xiaxl@bjfu.edu.cn (X.X.); 2 National Engineering Laboratory for Tree Breeding, Beijing Forestry University, Beijing 100083, China; 3 College of Biological Sciences and Technology, Beijing Forestry University, Beijing 100083, China

**Keywords:** autophagy, stress condition, transcription factor, phytohormone, TOR, SnRK1

## Abstract

To survive in stressful conditions, plants have developed multiple strategies to relieve damage. One of the strategies is to clear the damaged protein and organelles. Autophagy is a highly conservative degradation process, which refers to the recycling of damaged protein and organelles. Over the past decades, increasing evidence has revealed the important roles of autophagy in response to stress conditions, and many factors have been revealed involved in the sophisticated regulation of the autophagy signaling pathway. However, the accurate regulation pathway of the autophagy pathway is largely unknown. The current review proposes how stress-response factors respond to stress conditions involved in regulating the autophagy signaling pathway. In short, clarifying the regulating pathway of autophagy in response to stress conditions is beneficial to plant breeding.

## 1. Induction

Plants are sessile organisms and continuously encounter unfavorable growth conditions, including biotic and abiotic stress such as water deficiency, high temperature, cold, salt or pathogen infection [1]. To survive in these adverse environments, plants have developed many intricate strategies in offsetting the damage. Autophagy, a highly conserved self-eating process among various organisms, refers to recycling damaged proteins and specific compounds and activates some certain stress–response pathways [2]. Recently, autophagy has been regarded as one of the important mechanisms that help plants avoid harmful environments [3,4]. In plants, autophagy is classified into three types: macroautophagy, microautophagy and chaperone-mediated autophagy (CMA). As the name implies, macroautophagy means that it possesses an autophagic vacuole with a large volume, which is composed of double-membrane structure, used to package harmful or undesired cytoplasmic components and transport them to tonoplast for degradation [5]. Microautophagy is a pattern that tonoplast directly packages target substrates in the cytoplasm via its vacuole membrane invagination. Then, the packaged substrates are degraded for cyclic utilization. CMA is a specific process; it involves the development and pathogen-induced programmed cell death (PCD). During CMA, the changed permeability of the vacuole membrane results in the vacuole inclusion leaking out into the cytoplasm and the vacuolar hydrolases accelerate the degradation of cellular structure and programmed cell death (PCD) [5].

Autophagy, a complicated self-eating process, has been illuminated to be induced and involved in many aspects of plant growth, development and stress responses [2,6]. However, the knowledge of accurate regulatory networks during the process of autophagy in the cell is still limited. As we all know, there are multilevel signal pathways involve in the regulation of development and stress-responding [7,8]. In the present study, some factors which participate in the regulation of autophagy have been described and help us to understand the regulatory process during autophagy [2,6,9]. The current review will describe the recent advances of the regulatory factors involved in the modified the autophagy process in response to stress conditions.

## 2. TOR and SnRK1 Signal Network

**Figure 1:**
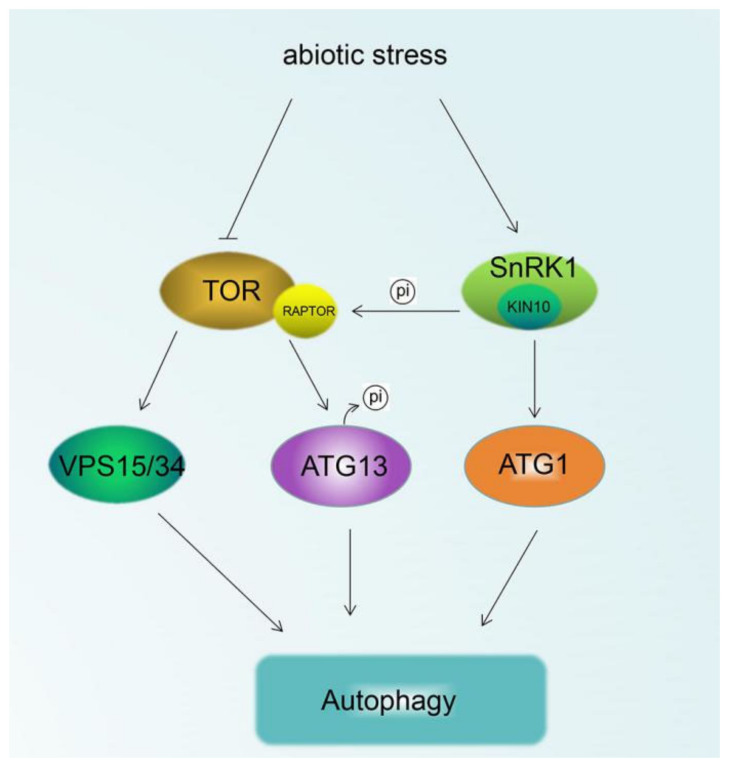
Effect of TOR and SnRK1 on plant autophagy in response to abiotic stress. Arrows show that the process is promoted, and the T-bars mean the process is inhibited. TOR: target of rapamycin; SnRK1: SNF-related kinase 1; ATG1/13: autophagy-related gene 1/13; RAPTOR: regulatory-associated protein of TOR; VPS15/35: vascular protein sorting 15/34.

The target of rapamycin (TOR) is a master regulator of nutrient factors during the process of plant growth and development. It functions as a member of the phosphatidylinositol-3 protein kinase [10,11]. The signal pathway of TOR has been elucidated in many reports for its roles in autophagy in yeast and animals [12,13]. Plants TOR has also been described in *Arabidopsis* for its role as a negative regulator of autophagy and nutrient sensor. For example, autophagy-regulated genes were triggered striking upregulation after treatment with TOR inhibitor (AZD8805) in *Arabidopsis.* These genes contain *VPS15*, *VPS34* (*PI3P*), *ATG7*, *ATG8*, *ATG9* and *ATG13*, which are pivotal genes for the formation of autophagosomes [14]. Similarly, decreased *TOR* expression in *RNAi-AtTOR* plants increased the transcription level of some *ATG genes* and the formation of autophagosomes suggesting that TOR is a negative regulator of autophagy [15]. Meanwhile, overexpression of *TOR* in *Arabidopsis* led to decreasing activity of autophagy during salt and osmotic stress [16]. ATG13 is the target of TOR during nutrient conditions. In *Arabidopsis*, the inactivation of TOR triggers dephosphorylation of ATG13, activating the origination formation of autophagosome [17]. A recent study reported that AtATG13 interacts with the regulatory-associated protein of TOR (AtRAPTOR) via a short amino-acid sequence (F-S-D-I-F) exhibited in the AtATG13 to regulate the autophagy system. The plants with the absence of the amino-acid F-S-D-I-F sequence in AtATG13 conferred an increased autophagy activity, accompanying with the decreased phosphorylation of the AtATG13 protein by TOR kinase [18] suggesting that ATG13 is the essential protein for TOR in regulating autophagy (Figure 1).

Sucrose-nonfermentation1-related protein kinase1 (SnRK1), a heterotrimeric complex, functions as a master regulator of life in response to energy and nutrients limits [19,20]. Plant SnRK1 is homologous with yeast Snf1 and mammalian AMPK, which has been reported to activate the autophagy system during various stress conditions [17,21]. In *Arabidopsis*, the catalytic subunit of AtSnRK1 contains three isoforms KIN10, KIN11 and KIN12. KIN10 takes charge of the major activity of the AtSnRK1 complex. Overexpression of *KIN10* in *Arabidopsis* activated the autophagy system and promoted the formation of the autophagosome. The activated autophagy conferred plants enhanced tolerance to carbon starvation stress and postponed leaf senescence. However, activation of autophagy by most abiotic stress was blocked in the *kin10* mutant. Meanwhile, the phosphorylation of ATG1 was increased in the *KIN10-OX* lines compared with *ATG1a*-OX and *wild type*(WT) lines during carbon starvation. Thus, these results suggested that SnRK1 participated in the induction of autophagy by increasing the phosphorylation of ATG1s in *Arabidopsis* [22]. Besides that, further evidence revealed the interaction between SnRK1 and TOR. The component RAPTOR of the TOR complex can interact with AtKIN10 in vivo and can be phosphorylated by AtKIN10 in vitro [23]. As previously reported, pharmacological inhibition or deficiency of RATOR in *Arabidopsis* all induced autophagy under abiotic stress, while a mutant *kin10* was unable to activate autophagy upon abiotic stress. However, autophagy was induced in mutant *kin10* and WT after treatment with TOR inhibitor AZD [24]. Similar results were also proven in WT and TOR transgenic plants after treatment with the SnRK1 activator AICAR [24]. Thus, the authors concluded that SnRK1 is upstream of TOR (Figure 1).

## 3. Phytohormone Crosstalk with Autophagy

Autophagy is a process of protein degradation involved in homeostasis, and some researchers reported the crosstalk between phytohormones and autophagy [25,26,27].

**Figure 2:**
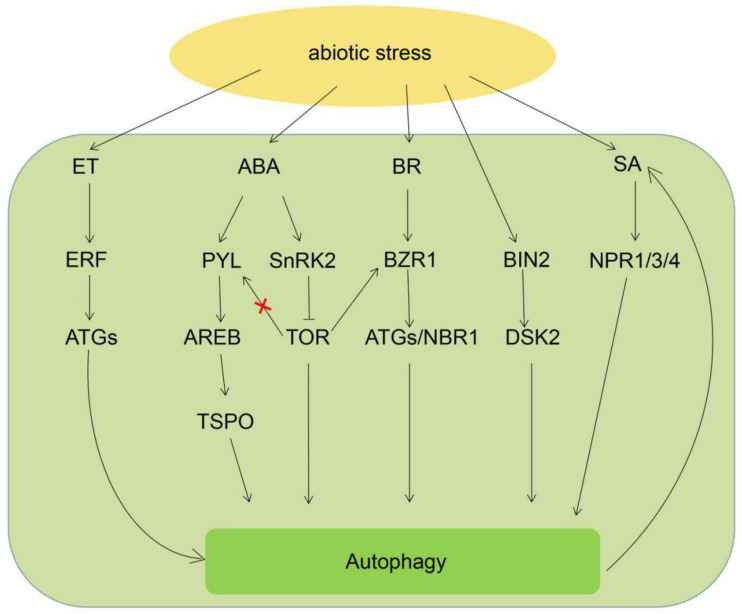
Effect of phytohormones on plant autophagy in response to abiotic stress. Arrows show that the process is promoted, and the T-bars mean the process is inhibited. ABA: abscisic acid; ET: ethylene; BR: brassinosteroids; SA; salicylic acid; SnRK2: SNF-related kinase 2; PYL: pyrabactin resistance-like; ERF: ethylene response factor; BIN2: BR insensitive 2; BZR1: brassinazole-resistant 1; NPR1/3/4: non-expresser pathogenesis-related protein1/3/4; AREB: ABA-responsive elements; DSK2: dominant suppressor of kar 2; NBR1: neighbor of BRCA1; TSPO: tryptophan-rich sensory protein.

Abscisic acid (ABA) is a major hormone accumulated under stress situations and participated in regulating plant stress tolerance [1]. The relation of ABA and autophagy is being explained in *Arabidopsis* as a model plant. Tryptophan-rich sensory protein (TSPO) is a member of the Trp-rich sensory protein/peripheral-type benzodiazepine receptor (TspO/MBR) protein family and a regulatory factor of various stress conditions and can be triggered by ABA and stress conditions. The *AtTSPO* is the downstream gene of the ABA signal pathway, which is directly conducted by the transcription factors abscisic acid-responsive element-binding proteins (AREBs) [28]. Heme and ATG8 interplay with TSPO, which contributes to the degradation of it via the autophagy system. The mutation of autophagy-regulated *atg5* resulted in inhibiting the degradation of AtTSPO in *Arabidopsis*, implying potential roles in autophagy during the ABA-dependent stress response [29]. Except that, ABA involves in the TOR pathway in regulating autophagy. In *Arabidopsis*, TOR functions as a kinase to catalyze the phosphorylation of pyrabactin resistance-like 1 (PYL1) in Ser119 under normal conditions, inhibit the activity of stress signaling, including the ABA signal pathway and promote plant growth. During stress conditions, ABA activates SnRK2, which promotes the phosphorylation of RATOR and inhibits the activity of the TOR complex. These reports revealed that ABA is a negative regulator of TOR under stress conditions and is involved in activating autophagy [30] (Figure 2).

Ethylene (ET) is another hormone plants use to respond to stress situations [31]. Some research has revealed links between autophagy and ethylene. In soybean plants, ACC (1-aminocyclopropane-1-carboxylate) treatment upregulated the transcription levels of *GmERF* and *GmATG8i*. Further research revealed that ACC pretreatment tomato exhibited higher activity of autophagy and increased expression levels of *ATG8d* and *ATG18h* compared with the control group under drought stress conditions. Furthermore, ERF5 can specifically interact with the promoters of *ATG8d* and *ATG18h* in vitro and in vivo, implying that the direct links between autophagy and ethylene [32]. ACC treatment of tomato enhanced the tolerance of drought stress, which was associated with the alternative oxidase-dependent autophagy.AOX-OE plants exhibited higher activity of autophagy and lower content of H_2_O_2_ than WT plants when pretreated with ACC, but it was adverse in AOX-silencing plants. Meanwhile, foliar treatment of H_2_O_2_ on the AOX-OE plants also exhibited lower autophagy activity in response to ACC while treatment *aox* mutant lines with the dimethylthiourea (DMTU, an H_2_O_2_ scavenger) compromised ROS generation whereas increased the autophagic activity [32]. These results implied that ET induced autophagy associated with the relatively low ROS signal improved AOX capacity, which scavenging ROS (Figure 2).

Brassinosteroids (BR) induced the interaction of brassinosteroid insensitive 1 (BRI1) with BRI1-associated receptor kinase 1 (BAK1) and BZR1, which active brassinazole-resistant 1 (BZR1) and BRI-emssuppressor 1 (BES1) involving in regulating downstream factors of BR signaling pathway [33]. Links between autophagy and BR signaling pathway have also been described in the plant. BR treatment triggered the upregulation of *ATG* genes and the formation of autophagosomes in tomato [34]. Autophagy activity in *dwf* mutant (BR deficiency lines) was detected lower than that of WT in tomato. However, autophagy activity was higher than that of WT plants in DWF-OE plants suggesting that BR is important in inducing the formation of autophagosomes. Meanwhile, researchers found that the absence of BZR1 protein in plants inhibited the formation of autophagosomes under BR treatment. So, the authors concluded that BZR1 is the key gene involving the BR-induced formation of autophagosomes. Further researches revealed that BZR1 directly interacts with the promoter of *ATG2*, *ATG6* and *NBR1* involves in the regulation of autophagy upon chilling stress. There are also researches showed that sugar signaling controls the accumulation of BZR1, promoting plant growth via regulating TOR [35]. BES1 is a major transcription factor responding to brassinosteroid (BR) pathway that regulating plant growth [33]. The degradation of BES1 is considered a ubiquitin-dependent manner. In *Arabidopsis*, AtBES1 is the target of DOMINANT SUPPRESSOR OF KAR 2 (DSK2). DSK2 is a selective autophagy receptor involved in the degradation of ubiquitinated proteins in plants. It also functions as a substrate of GSK3-like kinase brassinosteroid insensitive 2 (BIN2). The phosphorylation of DSK2 facilitates the interaction between DSK2 and ATG8. Following drought stress treatment, BES1 levels increased in *atg* or *dsk* mutant lines, while it decreased in WT lines [36]. Thus, autophagy is involved in the degradation of BES1 via DSK2-mediated autophagy system under stress conditions (Figure 2).

Salicylic acid (SA) is a signaling molecule involving the resistance of biotic stress in plants. The non-expresser pathogenesis-related (NPR) protein NPR1, NPR3 and NPR4 are very important for activating SA signaling pathway [37]. Recently, some researchers reported the crosstalk between SA and autophagy. In *Arabidopsis*, BTH (SA agonist) treatment induced the formation of autophagosomes. Moreover, autophagy deficiency lines (e.g., *atg2*, *atg5*) exhibited early senescence and pathogen-related programmed cell death (PCD) under normal conditions, and the content of endogenous SA is higher than that of WT plants. *NahG* encodes the SA hydroxylase to delete endogenous in *Arabidopsis*. Overexpression *NahG* in *atg2* or *atg5* inhibited the early senescence phenotype. Thus, the author considered that autophagy-induced early senescence is SA-dependent. Treatment of *NahG-atg5*, *NahG* and WT plants with SA agonist BTH recovered the early senescence phenotype. However, the early senescence phenotype was inhibited in the atg5-npr1 mutants indicating that the autophagy-deficiency-induced early senescence phenotype is dependent [38]. Further research revealed that NPR3 and NPR4 are also involved in the formation of the autophagosome. The *npr3* and *npr4* mutants accumulated much fewer autophagosomes and content of ATG7 and ATG8-PE [30]. Other research revealed that autophagy regulates the accumulation of SA. In apple, overexpression of ATG18a enhanced resistance in response to pathogen stress through promoting SA accumulation [39]. Similarly, SA biosynthesis genes are upregulated in *atg* mutant lines in *Arabidopsis* [40] (Figure 2).

## 4. Transcription Regulation of Autophagy

Transcription levels of *ATG* genes increased in response to the stress conditions [41,42]. However, the precise mechanism regulating the upregulation is largely unknown. In recent years, some transcription factors were proved to participate in the regulation of the autophagy system via directly binding to the promoter of *ATG* genes in *Arabidopsis* [43], tomato [34] and cassava [44].

**Figure 3:**
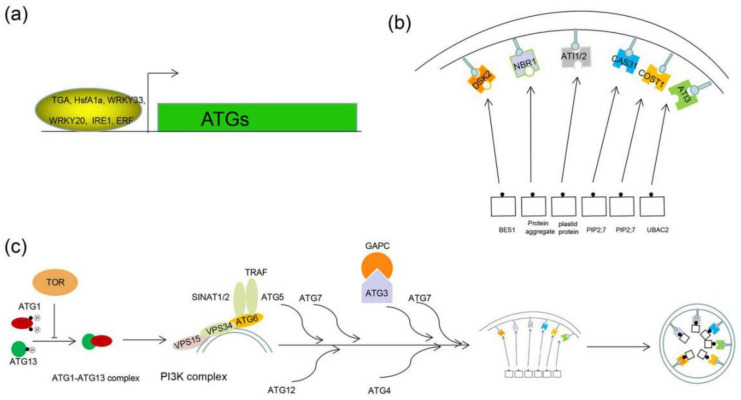
Autophagy pathway in plants. (**a**) Transcription factors involved in the regulation of autophagy in response to stress conditions; (**b**) the selective autophagy receptors interact with ATG8 and specific cargos; (**c**) the formation process of autophagosomes and the interaction proteins of ATGs. ATI1/2/3: ATG8 interacting protein1/2/3; BES1: BRI1-EMS suppressor 1; DSK2: domain suppressor of kar 2; UBAC2: ubiquitin-associated protein 2; NBR1: neighbor of BRCA1; TSPO: tryptophan-rich sensory protein or peripheral-type benzodiazepine receptor; TRAF: tumor necrosis factor receptor-associated factor; GAPC: cytosolic glyceraldehyde-3-phosphate dehydrogenase.

In *Arabidopsis*, Wang et al. [43] screened numerous transcription factors that may involve the regulation of *ATG8* via yeast one-hybrid system. Among them, TGA family members were proven to be involved in the upregulation of *ATG8b* and *ATG8e* in the protoplast. Overexpression of *TGA9* in *Arabidopsis* increased transcription level of *ATG1a*, *ATG3*, *ATG5*, *ATG8a/b/e/f/g*, *ATG13b and ATG18h* compared with WT plants. Under sugar starvation, the expression level of *ATG8b* increased in the *TAG9*-OE plants compared with WT. In the mutant, the TGA motif in the promoters of *ATG8B* and *ATG8E* completely abolished the binding between TGA9 and the promoters. These results suggested that TGA9 is the direct regulator of *ATG8* that involves the formation of autophagosome [43] (Figure 3b).

Hsf, a common thermal shock transcription factor, exists in almost all plants, positively regulates the stress-tolerance via interacting with HSP proteins [45]. Recent reports revealed *HsfA1A* was induced under drought stress and involved in the regulation of autophagy [46]. Silence *HsfA1a* triggered a sensitive phenotype to drought stress by increasing the accumulation of insoluble proteins. The contrary result was obtained from the *HsfA1a*-OE plants and the increase of the autophagy system. Chromatin immunoprecipitation analysis revealed that the *HsfA1a* is an upstream regulator of *ATG18f*, which is directly bidden with the promoter of it. Scilencing *ATG18f* resulted in suppressing the drought tolerance of *HsfA1a*-OE lines and inhibiting the formation of the autophagosome. Hence, the author considered the *HsfA1a* as a direct regulator of *ATG18f*, which contributes to promoting the formation of autophagosome under drought stress [46] (Figure 3a).

*WRKY* family members were found mainly in higher plants, functions as the regulator of plant defense, senescence and development [47]. In *Arabidopsis*, WRKY 33 is an interactor of ATG18a and involves in the induction of autophagy after inoculation infection with Botrytis. The expression level of *ATG18a* was induced after infection with *Botrytis* in 2, 3 and 4 dpi. However, it was similar to control after infection with *Botrytis* for 1 dpi, and it was barely detectable after infection for 3, 4 dpi in *wrky33* mutant lines suggesting that the roles of *wrky33* in the induction of *ATG18a* in the late of infection. Further researches indicated that *Botrytis* infection activates the autophagy system in the areas surrounding lesions in WT plants. Reduced autophagy activity was detected in the areas surrounding the lesion in *wrky33* and *atg18a* mutant after infection with *Botrytis* compared with WT plants [48], suggesting that *WRKY33* positively regulates the autophagy in pathogen-induced resistance. In tomato, heat stress activates the autophagy system in WT, *PTRV* (vector control lines) and *PTRV-WRKY33* (*WRKY33* silencing lines). However, the silence of *wrky33* significantly reduced the number of autophagosomes after heat stress [49]. Moreover, *MeWRKY20*, a homologous gene of *AtWRKY24*, was identified as the upstream regulator of *MeATG8a* involving plant disease resistance in cassava [44]. Overexpression of *MeWRKY20* upregulated the transcription level of *ATG8a*. At the same time, the expression level of *ATG8a* decreased in the *MeWRKY20*-silencing lines within the low activity of the autophagy system compared with control lines [44]. These results indicated that *WRKY* involves in the positive regulation of autophagy via directly interacting with the upstream promoter element of *ATG* genes (Figure 3a).

IRE1, a dual-functional protein, functions as protein kinase and ribonuclease, participating in RNA splicing [50]. In *Arabidopsis*, IRE1 consist of two members IRE1A and IRE1B has been considered as the sensor of ER stress and splices the transcription factor *BZIP60*. As knowns to all, autophagy is always activated upon ER stress and transports fragmented ER to vacuole to degradation [51]. ER-induced autophagy is linked with IRE1. In *ire1b* mutant lines, autophagy was not induced after the treatment of DTT or TM (ER stress agonists). At the same time, autophagy was elevated during DTT or TM treatment in both *ire1a* mutant and WT lines [52]. Starvation stress such as carbon or nitrogen stress elevated autophagy in *ire1a*, *ire1b and WT* lines suggesting that IRE1b is necessary for the ER stress-induced autophagy. In the *irea–ireb* mutant, overexpression of the RNAse-dead construct (a ribonuclease deficiency construct of IRE1B) did not rescue the ER-stress induced autophagy and the ER induced autophagy was rescued in the kinase-dead (a kinase deficiency construct of IRE1B) transgenic lines suggesting that the ribonuclease roles, but not protein kinase of IREB are necessary for the ER stress-induced autophagy [53] (Figure 3a).

## 5. The Core Autophagy Process and Proteins Interaction with ATG in Plants

The core autophagy process in plants composed of 18 proteins and belong to four parts of protein complexes (i) ATG1-ATG13 complex; (ii) ATG9 vesicle and ATG2-ATG18 complex; (iii) class III phosphatidylinositol-3-kinase (PI3K) kinase complex; (iiii) ATG8 and ATG12 Ubiquitination system complexes [10,54]. Under normal conditions, the target of rapamycin (TOR) kinase-dependent phosphorylation of the Atg1/Atg13 regulatory complex inhibits phagophore initiation While during stress conditions, the stress signal inactive the TOR inducing the ATG1-ATG13 complex participant the formation of pre-autophagosomal structure (PAS). Then, the PI3K complex recruits the ATG 18, ATG2, ATG9 complex for membrane elongation; the ATG12 and ATG8 conjugation complexes participate in the elongation, enclosure and anchoring of ATG8 into the membrane of the autophagosome. Finally, the autophagosome with cargos fused with vacuole [55] (Figure 3c).

A neighbor of BRCA1 (NBR1), a receptor of autophagy interacting with ATG8 via the AIM (ATG8 family interacting motif) domain during degrading some certain substrates in the cytoplasm, has been well studied in plants [34,56]. In *Arabidopsis*, the *nbr1* mutant exhibited a vulnerable phenotype in response to various stress conditions such as heat, oxidative, drought, salt and heat stress [56]. Similar results were also reported in the tomato. Chillings stress inhibited the degradation of BZR1, which promotes the upregulation of the *ATG* and *NBR1* genes resulting in activating autophagy. Silencing *ATG* and *NBR1* gene triggered the accumulation of ubiquitinated insoluble protein and decreased the numerous autophagosomes under cold stress suggesting that the important roles of *NBR1* in resisting stress conditions via autophagy pathway [34]. Moreover, the results in tobacco revealed that *nbr1* involves the response to sulfur deficit [57] (Figure 3b).

Tryptophan-rich sensory protein (TSPO) functions as a receptor of selective autophagy and was degraded via interacting with ATG8. Induction of TSPO decreased the level of plasma membrane intrinsic proteins 2;7 (PIP2;7) in *Arabidopsis* WT lines, but it was not changed in the TSPO-deficiency plants. Interestingly, the abundance of PIP2;7 still unaffected in the ATG5-deficiency lines suggesting the degradation of PIP2;7 required selective autophagy [58]. Moreover, CAS31 has been revealed as another receptor for degradation of PIP2;7. In *M. truncatula,* MtCAS31 can specifically interact with ATG8a involving the autophagy process [59]. Under normal conditions, the *CAS31* was barely detectable. Drought stress, however, significantly upregulated the expression level of *CAS31*. Overexpression of *CAS31* enhanced the tolerance of drought stress while the ability was decreased in the CAS31-deficiency lines compared with WT plants. Drought stress-induced accumulation of GFP signal in the membrane and nucleus at 4 h and partial GFP signal was detected in the vacuoles after 8 h of treatment in *MtCAS31pro:MtCAS31-GFP* lines. Concanamycin A (ConA) is a vacuolar proton pump inhibitor which is always used to block the autophagic degradation pathway. When ConA was added to block autophagy in *MtCAS31pro:MtCAS31-GFP*, punctate GFP fluorescence accumulated, suggesting that MtCAS31 is degraded via the autophagy pathway. Further analysis revealed that CAS31 could directly interact with PIP2;7 and regulated the degradation of it via ATG8a mediated autophagy pathway. Therefore, the author regarded CAS31 as a new receptor for selective autophagy [59] (Figure 3b).

Constitutively stressed 1 (COST1), a plant-specific protein, was first identified in *Arabidopsis* because of the roles of protein in regulating stress tolerance [60]. The *cost1* mutant up-related the gene set associated with drought, salt and cold stress exhibiting the enhanced phenotype of drought stress. Further research revealed that COST1 could not only colocalize with ATG8e and NBR1 but also interact with ATG8e, implying that COST1 plays an important role in selective autophagy. In addition, upon treatment of ConA, the COST1-YFP (cost1-1 mutant background, with full complementation) lines enhanced the accumulation of ubiquitinated COST1. *cost*-*atg5* and *cost1*-*atg7* mutants exhibited the sensitivity to drought stress, which is similar to the *atg5* and *atg7* mutants suggesting that *cost1* functions as the upstream factor of autophagy. Overexpression of *COST1* reduced the constant of *ATG8e* in the proteins level, but not the transcription level suggesting that *COST1* mediated decrease of ATG8e is post-transcriptional [60] (Figure 3b).

ATG8 interacting protein1/2 (ATI1 and ATI2) are specific in plants regarding as a typical autophagy cargo receptor [61,62]. Under normal conditions, they are related to the endoplasmic reticulum membrane network. Upon carbon starvation, however, ATI1/2 is relevant to ER-related bodies and plastids-related bodies [62]. In terms of ER-related bodies, carbon starvation-induced the formation of spherical compartments, which contain ATI1, moves along with the ER network and delivery to the vacuoles. In terms of plastids-related bodies, ATI1, located in the ATI1-PS bodies (ATI1-contain plastids-associated bodies), where it interacted with plastid-related proteins and ATG8 involved in their cycling in vacuoles, suggesting that ATI1 and ATI2 may function in the ATG8-dependent proteolysis [63] (Figure 3b).

ATG8 interacting protein 3 (ATI3) is another kind of interacting protein of ATG8, which is found only in dicots but not in other plants such as monocots [64]. In *Arabidopsis*, there are 3 ATI3A/B/C proteins, and it interacts with ATG8 via the WXXL (LC3-interacting region, LIR) motif existed at the C terminus. The roles of ATI3 are mainly related to its interaction with ATG8. In *Arabidopsis*, the *ati3* mutant is sensitive to heat stress and pathogen stress. Overexpression of the wild-type *ATI3A* coding sequence in *ati3a* mutant restored the tolerance of heat stress, while overexpression of the *AT3I^W260A^*, which was changed the tryptophan residue at amino acid position 260 of ATI3A in the canonical WxxL LIR motif into an alanine residue, in *ati3a* mutant did not alter the tolerance of heat stress suggesting that ATI3A confers plant abiotic stress tolerance via interacting with ATG8. ATI3A can also interact with ubiquitin-associated protein 2 (UBAC2), which is localized in the ER and is implicated in endoplasmic reticulum stress. Overexpression of UBC promotes the interaction of ATI3 and ATG8, increasing the formation of autophagosomes. However, the *ati3* and *ubac2* mutants are observably accommodationist in response to the treatment of ER-stress. Interestingly, autophagy-dependent ER degradation was not damaged. Thus, the author proposed that ATI3 interact with UBC2, play important roles in response to stress conditions by regulating selective autophagy of ER components [64] (Figure 3b).

Cytosolic glyceraldehyde-3-phosphate dehydrogenase (GAPC) is revealed to transduce H_2_O_2_ signal under stress conditions, and it can interact directly with H_2_O_2_ involved in ROS response [65]. In recent years, GAPC was also clarified to participate in the regulation of the autophagy system by interacting with ATG3 in tobacco. Methyl viologen (MV) is a well-known oxidative stress inducer in plants. The treatment of MV inhibited the interaction between GAPC and ATG3 in tobacco. Silencing of *GAPC* activated autophagy. However, overexpression of *GAPC* inhibited the formation of autophagosomes. Thus, the GAPC is a negative regulator of autophagy [64] (Figure 3c).

Tumor necrosis factor receptor-associated factor (TRAF) family proteins regulate autophagy via interacting with ATG6 [66]. In *Arabidopsis*, the double mutant traf1a-b showed premature senescence phenotype under carbon and nitrogen starvation, while the complemental lines TRAF1A/traf1a/b completely rescued the sensitivity to starvation stress. In addition, TRAF1a and TRAF1b were induced and located in autophagosomes during carbon starvation. Autophagosomes accumulation in double mutant traf1a-b lines is less than that of WT lines during nitrogen and sucrose starvation [66]. ATG6 is a vital component during the process of autophagosome formation [67]. The posttranslational ubiquitination of ATG6 is involved in determining the levels of autophagy. In double mutant traf1a/b, the ubiquitination level of ATG6 is lower compared with WT lines. *Arabidopsis* ATG6 is the direct target of SINATs and ubiquitinated by SINAT1 and SINAT2. TRAF1a and TRAF1b are required for SINAT1-and SINAT2-associated ubiquitination and the degradation of ATG6 in plants [66] (Figure 3c).

## 6. Future Perspectives

This review article summarizes the recent research on the knowledge of plant autophagy and its regulatory mechanisms in response to abiotic stress, including drought, chilling, heat, salt and starvation stress. Many researchers have indicated that autophagy is essential to coping with stress conditions, and the regulatory mechanism of autophagy is complicated, which refers to multilevel factors such as transcription factors, phytohormone, interacting proteins, TOR, SnRK1, and so on. Though some research revealed some of the factors involved in regulating autophagy under stress conditions, there are still many open questions regarding the exact mechanism. A subject that is often in the spotlight in recent years is selective autophagy. At present, several autophagy receptors associated with the stress response have been identified. There are still many receptors—and the specific substrates of the receptors—that need to be identified. According to the present study, selective autophagy receptors contain the specific domain AIM. Therefore, bio-information and transcriptome analysis will be useful for the identification of the candidate receptors. Then, the yeast two-hybrid, bimolecular fluorescence complementation (BiFC), pull-down, and co-immunoprecipitation (CO-IP) will be the effective methods for verifying the interactions between candidate receptors and ATG8. In addition, the candidate receptor mutant and overexpression line will be useful for analyzing their biological function. The receptors and substrates help us better understand the mechanism that plants perceive and adapt to stress conditions. Another important issue is which and how transcription factors involve in regulating autophagy during stress conditions. Although several transcription factors in the different pathways have been revealed to regulate autophagy, our knowledge of it remains limited. The yeast one-hybrid and CHIP-sequence are still effective ways of selecting the upstream promoter of ATGs. The last issue is that phytohormone is a set of important regulator factors in response to stress conditions. However, the effect of phytohormone on the regulation of autophagy is still largely unknown. For example, current research revealed that ABA is involved in the autophagy process via TOR or TSPO pathway, but if there are additional ways for the ABA to involve in the autophagy system is still needed further study. Genetic methods such as signal, double or triple mutants are useful materials for identifying the relationships between autophagy and phytohormone.
